# Cannabidiol as a Potential Treatment for Anxiety and Mood Disorders: Molecular Targets and Epigenetic Insights from Preclinical Research

**DOI:** 10.3390/ijms22041863

**Published:** 2021-02-13

**Authors:** Philippe A. Melas, Maria Scherma, Walter Fratta, Carlo Cifani, Paola Fadda

**Affiliations:** 1 Center for Psychiatry Research, Department of Clinical Neuroscience, Karolinska Institutet & Stockholm Health Care Services, 11364 Stockholm, Sweden; philippe.melas@ki.se; 2 Center for Molecular Medicine, L8:00, Karolinska University Hospital, 17176 Stockholm, Sweden; 3 Division of Neuroscience and Clinical Pharmacology, Department of Biomedical Sciences, University of Cagliari, 09042 Cagliari, Italy; wfratta@unica.it (W.F.); pfadda@unica.it (P.F.); 4 Pharmacology Unit, School of Pharmacy, University of Camerino, 62032 Camerino, Italy; carlo.cifani@unicam.it; 5 CNR Institute of Neuroscience-Cagliari, National Research Council, 09042 Cagliari, Italy

**Keywords:** cannabidiol, anxiety, depression, 5-HT1A receptors, TRPV1 receptors, CB1 receptors, DNA methylation, histone modifications, miRNA, epigenetics

## Abstract

Cannabidiol (CBD) is the most abundant non-psychoactive component of cannabis; it displays a very low affinity for cannabinoid receptors, facilitates endocannabinoid signaling by inhibiting the hydrolysis of anandamide, and stimulates both transient receptor potential vanilloid 1 and 2 and serotonin type 1A receptors. Since CBD interacts with a wide variety of molecular targets in the brain, its therapeutic potential has been investigated in a number of neuropsychiatric diseases, including anxiety and mood disorders. Specifically, CBD has received growing attention due to its anxiolytic and antidepressant properties. As a consequence, and given its safety profile, CBD is considered a promising new agent in the treatment of anxiety and mood disorders. However, the exact molecular mechanism of action of CBD still remains unknown. In the present preclinical review, we provide a summary of animal-based studies that support the use of CBD as an anxiolytic- and antidepressant-like compound. Next, we describe neuropharmacological evidence that links the molecular pharmacology of CBD to its behavioral effects. Finally, by taking into consideration the effects of CBD on DNA methylation, histone modifications, and microRNAs, we elaborate on the putative role of epigenetic mechanisms in mediating CBD’s therapeutic outcomes.

## 1. Introduction

Cannabidiol (CBD) was first isolated from the Cannabis plant in 1930, and its chemical structure was characterized in 1940 [1]. CBD is the second major compound present in *Cannabis sativa* and, unlike ∆9 -tetrahydrocannabinol (THC), CBD does not have any psychotomimetic activity. In contrast to THC, CBD displays very low affinity for cannabinoid 1 and 2 (CB1, CB2) receptors [2]. Moreover, compared to THC, CBD presents with a much better safety profile. Specifically, studies on the safety and tolerability of CBD demonstrate no significant side effects with oral doses up to 1500 mg/day or with intravenous doses of 30 mg [3]. However, the pharmacodynamic profile of CBD appears to be complex, and the mechanisms of action are not yet completely understood. Specifically, it is thought that CBD exerts its molecular and behavioral effects through various molecular targets. For example, CBD can inhibit fatty acid amide hydrolase (FAAH), preventing the catabolism of the endogenous cannabinoid anandamide (AEA) [4,5]. CBD can also act as an allosteric modulator of the serotonin type 1A (5-HT1A) receptor, promoting the agonist-related stimulation of GTPgammaS binding [6]. Moreover, CBD was found to activate and desensitize the transient receptor potential cation channel subfamily V members 1-2 (TRPV1-2) [7]. Other studies suggest that CBD may also (i) act on adenosine receptors [8], (ii) inhibit the synaptosomal uptake of monoamines and γ-aminobutyric acid GABA [9], and (iii) activate the expression of peroxisome proliferator-activated receptor (PPAR) gamma [10]. Since CBD interacts with a wide variety of molecular targets with multiple mechanisms of action, CBD’s therapeutical potential has been investigated in many diseases and pathological conditions such as inflammation, neuropathic pain, and epilepsy [11]. In addition, various independent studies support the view that CBD could represent a new approach to treating anxiety and mood disorders [12,13]. In this regard, CBD has been tested in several animal models of anxiety and depression showing promising results in decreasing anxiety and improving depressive-like behaviors [14]. However, the molecular mechanisms underlying these therapeutic-like effects are just beginning to be understood. In the present review article, we first provide an overview of animal-based studies that evaluated CBD as an anxiolytic- or antidepressant-like compound. Next, we describe CBD’s neuropharmacological profile in the context of its anxiolytic- or antidepressant-like properties. Finally, we elaborate on CBD’s putative epigenetic mechanism of action by reporting studies that examined how CBD affects DNA methylation, histone modifications, and microRNA expression.

## 2. Methods

To find literature on the behavioral and neuropharmacological effects of CBD, a PubMed database search was performed using the combination of the following keywords: cannabidiol, psychiatric disorders, anxiety disorders, depression, cannabinoid receptor, 5-HT1A receptor, and TRPV 1 receptor. From the search results we included animal studies only. For literature available on the epigenetic effects of CBD, PubMed searches were conducted using the combination of the term ‘cannabidiol’ with the following keywords: epigenetics, histone, methylation, hydroxymethylation, microRNA, and noncoding RNA. The search results were again evaluated for relevance in the context of the present review.

## 3. Anxiety Disorders

### 3.1. Behavioral Studies of CBD’s Anxiolytic-Like Properties

The anxiolytic effects of CBD have been highlighted in several animal models of anxiety (Table 1). The elevated plus maze (EPM) is a widely used experimental method to test anxiety-related behaviors in rodents, which is based on the conflict between the natural spontaneous exploratory behavior of rodents in novel environments and their natural aversion for open spaces [15]. Using this paradigm, it is possible to detect both anxiogenic- and anxiolytic-like property of drugs. In the EPM, acute systemic administration of CBD has been found to elicit, in both rats and mice, an inverted U-shaped dose–response curve, with anxiolytic effects at doses of 2.5, 5, 10 mg/kg, i.p., and 0.50, 1, 2.5, 5, 10, 50 mg/kg, i.p., respectively [16,17]. The lowest effective acute dose of 1 mg/kg (i.p.) of CBD was also found to exhibit anxiolytic properties when evaluated in the social interaction (SI) test, in which rats are placed in pairs in a test arena to measure their sociability, e.g., sniffing and grooming [18]. Moreover, using the Vogel’s conflict test (VCT), water-deprived rats treated with CBD (10 mg/kg, i.p.) accepted significantly more electric shocks during the conflict between drinking or being punished, thus reinforcing CBD’s anxiolytic profile [19]. Since stress is an important contributor to anxiety disorders, CBD has also been evaluated in stress-induced anxiety models. For example, CBD (10 mg/kg, i.p.) attenuated the increased anxiety behavior caused by previous exposure to acute restraint stress in rats [20]. Moreover, a low dose of CBD (5 mg/kg, i.p.) reduced anxiogenic responding in rats, produced by foot shock stress given 24 h prior to the light–dark emergence test [21]. The anxiolytic effects of CBD were highlighted even when CBD was directly injected in specific brain regions involved in the modulation of anxiety-like responses. Specifically, local administration of CBD (15–60 nmol/µL) into the dorsal portions of the periaqueductal gray matter (dPAG) of rats, produced anxiolytic-like effects in the EPM test with a “bell-shaped” dose–response curve with anxiolytic effects at doses of 30 nmol/µL [22]. Moreover, intra-dPAG administration of CBD (30 and 60 nmol/µL) also impaired inhibitory avoidance acquisition and inhibited the escape response in two proposed animal models of panic: the elevated T-maze (ETM) and the electric stimulation of the periaqueductal gray matter [23]. Additional results from studies carried out using the EPM and VCT tests in rats showed anxiolytic effects when CBD was microinjected into the central nucleus of the amygdala (1 µg/µL) [24], as well as into the bed nucleus of the stria terminalis (BNST) (30 nmol/µL) [25]. Microinjections of CBD (30 and 60 nmol/µL) into the BNST of rats also attenuated the expression of contextual fear conditioning (CFC) [26]. Finally, intracisternal administration of CBD (30 nmol/µL) decreased the anxiogenic consequences of restraint stress in rats [27]. On the other hand, intra-prelimbic (PL) prefrontal cortex injection of CBD (30 nmol/µL) in rats was either anxiogenic or anxiolytic depending on the animal model used (EPM or CFC, respectively) [28].

### 3.2. Neuropharmacological Studies of CBD’s Anxiolytic-Like Properties

The pharmacodynamic profile of CBD appears to be complex, since it acts on various molecular targets including cannabinoid, TRPV1, and 5-HT1A receptors [6,7]. Moreover, CBD facilitates endocannabinoid signaling by inhibiting the hydrolysis of AEA [4,5]. All these molecular targets have been studied to evaluate their involvement in the anxiolytic effects of CBD [33]. For instance, the acute anxiolytic effects of CBD injected either systemically (10 mg/kg, i.p.) or into specific brain areas (30 nmol/µL) were found to be abolished by the 5-HT1A receptor antagonist WAY-100635, suggesting that activation of these serotonin receptors underlies CBD’s anxiolytic effects [20,22,23,25,28]. The 5-HT1A receptors are widely distributed in brain areas related to stress and anxiety, such as the prefrontal cortex, hippocampus, and amygdala [34]. Moreover, agonists and partial agonists of these receptors exert anxiolytic activity in various animal models of anxiety [35]. Also, ablation of the gene encoding the 5-HT1A receptor leads to the generation of knockout (KO) mice with a strong anxiety-like behavioral phenotype [36,37]. The molecular mechanism by which CBD activates 5HT1A receptors still needs to be clarified, although there is evidence suggesting that it may act as an allosteric modulator increasing [35S] GTPγS binding [6]. On the other hand, however, intra-dPAG administration of the dose of CBD (60 nmol/µL) that alone evokes no effect together with the TRPV1 antagonist capsazepine was found to increase open-arm exploration in the EPM test [29]. This suggests that the activation of TRPV1 receptors may be responsible for either no effects or anxiogenic effects at high doses. TRPV1 receptors are expressed in various brain regions related to anxiety, including the periaqueductal gray matter where their activation can increase glutamate release and thus facilitate anxiety responses [29]. In line with this assumption, TRPV1 KO mice exhibit less anxiety-related behavior in the light–dark and in the EPM tests compared to their wild-type littermates [38]. With regard to cannabinoid receptors, administration of the CB1 selective antagonist AM251 failed to prevent the anxiolytic effects of CBD injected into the periaqueductal gray matter [22]. Although most studies mentioned so far mainly concern the acute effects of CBD, some studies have also investigated the anxiolytic effects of CBD after chronic treatment. One such study found that repeated CBD treatment (5 mg/kg/daily/21 days) in rats inhibited the escape response evoked in the ETM test, an anti-panic effect that appeared to rely on the direct activation of 5-HT1A receptors located in the dPAG, since the effect was blocked by intra-dPAG injections of WAY100635 [30]. Another study found that repeated CBD treatment (5 mg/kg/daily/7 days) was able to prevent anxiety-like behaviors in rats experiencing neuropathic pain, with 5-HT1A receptors again mediating this behavioral effect [39] and with CBD being able to rescue the impaired 5-HT neurotransmission in these animals [39]. Chronic CBD administration (30 mg/kg, i.p., 2 h after each daily stressor/14 days) has also been found to reduce anxiety in mice exposed to chronic stress by facilitating hippocampal neurogenesis. In this case, however, the effects of CBD were reversed by AM251, suggesting an involvement of increased endocannabinoid tone in CBD’s anxiolytic effects [31]. Finally, it should be mentioned that although most preclinical studies support an anxiolytic role for CBD, chronic administration of CBD (10 mg/kg/daily/14 days) has also been reported to produce anxiogenic-like behaviors, e.g., in rats subjected to a conditioned emotional response (CER) [32]. This effect was associated with a reduction in brain-derived neurotrophic factor (BDNF) expression in the hippocampus and frontal cortex as well as with a reduction in tyrosine kinase B (Trk B) receptor expression in the hippocampus only [32].

### 3.3. Putative Epigenetic Mechanisms Underlying CBD’s Anxiolytic-Like Properties

Although the exact mechanisms responsible for CBD’s anxiolytic effects remain poorly understood, it has been hypothesized that epigenetic modifications may mediate CBD’s effects on psychiatric phenotypes [40]. Epigenetics refers to mechanisms that can alter gene expression without altering the underlying DNA sequence. The most studied epigenetic mechanisms include DNA methylation and histone modifications—and their interplay [41]—as well as non-coding RNA-associated gene silencing through microRNAs [42]. Epigenetic changes have been found to contribute to psychiatric disorders, including anxiety and mood disorders [43,44]. Moreover, commonly used pharmacological agents for depression, bipolar disorder, and anxiety have been shown to induce epigenetic changes that are associated with remission [45,46,47]. Epigenetic mechanisms also represent a way by which the environment can affect gene activity independently of the underlying DNA sequence. This is illustrated by early-life stress, a common risk factor for anxiety and mood disorders, which has been linked to aberrant epigenetic changes in key genes involved in regulating the hypothalamic–pituitary–adrenal axis response [48].

#### 3.3.1. CBD and DNA Methylation

The majority of DNA methylation occurs through the covalent attachment of a methyl group to the C5 position of cytosine at cytosine–guanine dinucleotide (CpG) residues, which is catalyzed by maintenance or de novo DNA methyltransferases (DNMTs) [49]. DNA methylation at promoter and first-exon gene regions is most often coupled to decreased levels of gene expression [50], whereas global DNA methylation levels are associated with overall genomic integrity [51]. The first known epigenetic study of CBD examined its effect on DNA methylation in human keratinocytes (HaCaT cells), given the endocannabinoid system’s role in controlling skin physiology and differentiation [52]. In the latter study, CBD (0.5 μM) was found to have two effects on DNA methylation: first, it increased gene-specific DNA methylation of epidermal differentiation-related genes, such as the keratin 10 gene, which was accompanied by concomitant reductions in gene expression [52]; second, CBD caused an increase in the levels of global DNA methylation, which was accompanied by increased expression of the maintenance DNA methyltransferase Dnmt1 [52]. However, subsequent studies examining how CBD affects DNA methylation in the brain have uncovered a more complex picture, which illustrates the importance of the tissue under investigation in epigenetic analyses. For instance, using a rodent model of brain iron loading, CBD normalized the methylation levels of mitochondrial DNA in the hippocampus [53]. Specifically, iron administration during the neonatal period induced significant decreases in mitochondrial DNA methylation in the hippocampus of adult rats, an outcome that was reversed in adulthood by CBD treatment (10 mg/kg, i.p.) for 14 consecutive days [54]. Moreover, and highly relevant to the present review, recent studies have examined the effects of CBD on nuclear DNA methylation in the rodent hippocampus [54]. Specifically, hippocampi from adult wild-type mice that received CBD orally (20 mg/kg) for two weeks were found to have a skew toward global DNA hypomethylation, including hypomethylation of the de novo methyltransferase Dnmt3a and >3000 additional differentially methylated loci enriched for genes involved in neuronal function and synaptic organization [55]. CBD’s effect on the methylation status of Dnmt3a is of particular interest, since the expression of this de novo methyltransferase in the prefrontal cortex has been linked to anxiety-like behaviors in adult mice [55]. Specifically, increased anxiety-like behaviors have been found to be associated with a reduction in Dnmt3a mRNA levels and global DNA hypomethylation in the adult mouse prefrontal cortex [55]. Importantly, overexpression of Dnmt3a in the latter preclinical model produced anxiolytic effects [55]. These preliminary findings raise the possibility that the observed anxiolytic effects of CBD may rely on the action of DNMTs that restore DNA methylation levels at aberrantly regulated genes contributing to anxiety disorders.

#### 3.3.2. CBD and Histone Modifications

There is accumulating evidence suggesting that DNA methylation works in concert with histone modifications to regulate gene expression [41]. Histone modifications refer to covalent post-translational modifications of histone proteins. Over a hundred of these modifications have been described in the literature, including acetylation, methylation, phosphorylation, ubiquitylation, and sumoylation [56]. Histone modifications are catalyzed by a number of histone-modifying enzymes, such as histone acetyltransferases and histone deacetylases, which can affect gene expression by altering the chromatin structure. The effect of histone modifications on gene expression is not uniform but depends on parameters such as the type of modification and the amino acid residue of the histone protein that is being modified [57]. For instance, tri-methylation of the histone 3 lysine 4 residue (H3K4me3) in promoter regions is associated with transcriptional activation, whereas tri-methylation at histone 3 lysine 27 (H3K27me3) is associated with transcriptional repression. Studies in mouse models of multiple sclerosis have found that CBD (10 mg/kg, i.p.) can affect the levels of both H3K4me3 and H3K27me3 at specific genes, such as IL-4, IL-5, and IL-13, in splenic CD4^+^ T cells [58]. More relevant to the present review, however, are studies in brain tissue that have provided evidence that CBD can act synergistically with other compounds to induce histone hyperacetylation in the mesolimbic system [59]. Specifically, repeated co-administration of CBD and THC for 15 days (at a 5:1 CBD/THC ratio, i.e., 50 mg/kg/10 mg/kg, i.p.), but not the administration of either compound on its own, resulted in significantly increased levels of the gene-activating histone acetylation marks H3K9ac and H3K14ac in the ventral tegmental area of adult male mice [59]. However, similar to DNA methylation, the effects of exogenously administered compounds on histone modifications can vary from tissue to tissue and even among different brain regions. This was evidenced by a recent study that examined the levels of five different histone modifications (H3K4me3, H3K9ac, H3K9me2, H3K27me3, and H3K36me2) in the cerebral cortex, hypothalamus, and pons following the systemic administration of CBD (20 mg/kg, i.p.) to adult rats [60]. In the cerebral cortex, CBD enhanced H3K4me3, H3K9ac, and H3K27me3 levels, without producing any significant differences in H3K9me2 or H3K36me2 levels [60]. In the hypothalamus, CBD lowered H3K9ac levels, without producing any significant effects on H3K4me3, H3K9me2, H3K27me3, and H3K36me2 levels [60]. Finally, in the pons, CBD reduced H3K4me3 levels, without any significant effects on the levels of H3K9ac, H3K9me2, H3K27me3, or H3K36me2 [60]. The histone modifications affected by CBD have been linked to anxiety- and stress-related characteristics in separate preclinical studies. For example, a comprehensive study on the effects of stress that examined hippocampal H3K4, H3K9, and H3K27 methylation levels, found evidence that stress duration impacts histone modifications in a complex way [61]. Specifically, acute stress increased H3K9me3 but lowered H3K27me3 and H3K9me1 levels in the hippocampus [61]. By contrast, a week of restraint stress increased H3K9me3 but lowered H3K27me3 and H3K4me3 levels in the same region [61]. Thus, it is tempting to speculate that the observed stress-induced reductions in the hippocampal levels of H3K27me3 and H3K4me3 following a week of restraint stress [61] may be reversed by CBD’s enhancing effects on these same modifications, as reported in the cerebral cortex [60]. A close relationship is also known to exist between H3K27me and de novo DNA methylation [41], thus providing a possible link between CBD’s effects on histone modifications and the previously discussed effects of CBD on DNA methylation [54]. Another interesting anxiety-related epigenetic candidate is H3K9ac, whose levels were elevated in the cerebral cortex by CBD administration [60]. Low levels of H3K9ac in the central amygdala have been found to contribute to the maintenance of chronic anxiety and pain [62]. Decreased levels of H3K9ac have also been found at the glucocorticoid receptor gene (*Nr3c1*) in rats that show increased stress reactivity and anxiety-related behavior in adulthood as a result of receiving low levels of maternal care over their first week of life [63]. Thus, more systematic future studies are needed to examine the effect of CBD on histone modifications at key genes and in brain regions involved in the pathophysiology of anxiety.

#### 3.3.3. CBD and miRNAs

Coordinated actions of epigenetic mechanisms, such as DNA methylation and histone modifications, have also been reported in relation to microRNAs (miRNAs). MiRNAs are small non-coding RNAs that can suppress gene expression by binding to their target mRNA molecules [64]. The effect of CBD on miRNA expression has been investigated in different experimental models. For instance, in splenic CD4^+^ T cells from mouse models of multiple sclerosis, CBD treatment (10 mg/kg, i.p.) affected miRNAs in both directions, i.e., it increased the expression of certain ones and decreased the expression of others [58]. However, of particular relevance to the present review is the reported effect of CBD on miRNA expression in both resting and lipopolysaccharide (LPS)-activated microglial (murine BV-2) cells [65]. Microglia are the brain’s resident immune cells and have been implicated in a number of psychiatric disorders, including anxiety and mood disorders, with documented roles in synaptic pruning and neurogenesis [66]. In microglial BV-2 cells, pretreatment with CBD (10 μM) inhibited the LPS-stimulated expression of a redox-sensitive miRNA, miR-155, which regulates Nrf2-driven gene expression [65]. Interestingly, miR-155 knockout mice have been found to exhibit reduced anxiety-like behaviors [67], and Nrf2 has been found to be involved in the development of anxiety-like behaviors [68]. Moreover, in the BV-2 microglial study, CBD enhanced the expression levels of miR-34a and miR-449a [65], both of which have been implicated in anxiety-related traits. Specifically, a critical function for miR-34 in regulating the response to stress has been demonstrated using knockout mouse models, with miR-34a decreasing Notch signaling in the basolateral amygdala to facilitate fear memory consolidation [69,70]. Moreover, restraint stress in rats elevated the expression of miR-449a in the anterior pituitary, and miR-449a overexpression decreased corticotropin-releasing factor type 1 receptor (CRF-R1) expression [71]. Collectively, by affecting miRNA expression in addition to other epigenetic mechanisms, such as DNA methylation and histone modifications, CBD may modulate the stress response and exert anxiolytic effects. However, future studies are warranted to examine the causal role of these CBD-induced epigenetic modifications in ameliorating anxiety-related characteristics.

## 4. Mood Disorders

### 4.1. Behavioral Studies of CBD’s Antidepressant-Like Properties

The potential of CBD to reduce depressive-like behavior has been highlighted in several animal models of depression (Table 2). Results from the forced swimming test (FST), a rodent model in which animals are subjected to an inescapable stress and typically respond with alternating bouts of escape-oriented behavior and immobility, showed that a single injection of CBD (30 mg/kg, i.p.) induced antidepressant-like effects in mice, comparable to those of imipramine, a tricyclic antidepressant, and fluoxetine, a selective serotonin reuptake inhibitor [72,73]. In addition, a sub-effective dose of CBD (7 mg/kg, i.p.), when co-administered with a sub-effective dose of fluoxetine (5 mg/kg, i.p.), resulted in significant effects, thus implicating synergistic and/or additive mechanisms [73]. The antidepressant-like effects of CBD in the FST have been further confirmed in other studies, after both acute and chronic treatment at different doses (30 mg/kg, i.p. and 200 mg/kg, i.p.) [74,75]. Furthermore, the efficacy of CBD following acute (50 mg/kg, i.p.) and chronic administration (50 mg/kg/daily/3 days + 10 mg/kg/daily/11 days), was also highlighted in the olfactory bulbectomy mouse model of depression (OBX), which is characterized by behavioral phenotypes as well as anatomical, cellular, and biochemical changes similar to those observed in depressed patients [76]. CBD (30 mg/kg, orally) also showed positive responses in two genetic rat models of depression, the Wistar Kyoto (WKY) and the Flinders Sensitive Line (FSL) rats, which present with a number of behavioral and physiological endophenotypes that are often present in major depressive disorder [77,78].

### 4.2. Neuropharmacological Studies of CBD’s Antidepressant-Like Properties

The antidepressant-like effects of CBD, in both the FST test (30 mg/kg, i.p.) and the OBX model (50 mg/kg, i.p.), were found to be inhibited by pre-treatment with WAY100635, suggesting that CBD’s effects may depend on the activation of 5-HT1A receptors [72,76]. In agreement with this assumption, in vivo microdialysis studies showed that the administration of CBD (50 mg/kg, i.p.) significantly enhanced extracellular 5-HT and glutamate levels in the ventromedial prefrontal cortex (vmPFC) of OBX rats and these neurochemical effects were prevented by 5-HT1A receptor blockade [76]. Moreover, adaptive changes in pre- and post-synaptic 5-HT1A receptor functionality were also found after chronic CBD (50 mg/kg/daily/3 days + 10 mg/kg/daily/11 days) administration [76]. Chronic treatment with CBD (30 mg/kg/daily/14days, i.p.) increased amygdala BDNF levels in rats subjected to the FST [75]. In addition, the acute antidepressant effects of CBD (7–30 mg/kg, i.p.) in mice subjected to the FST were accompanied by increased BDNF levels in the hippocampus and medial prefrontal cortex (mPFC), as well as by increased spine density in the mPFC [79]. It has repeatedly been found that serum BDNF levels are lower in depressed patients and that antidepressant drugs increase BDNF expression and protein levels in both animals and humans [81,82]. However, Zanelati and collaborators failed to detect any effect of CBD (30 mg/kg, i.p.) on hippocampal BDNF levels in mice subjected to the FST [72]. Antidepressant effects in rats subjected to the FST were also evident when CBD (45 and 60 nmol/µL) was injected into the vmPFC, a brain region which plays a significant role in the stress responses [80]. These effects were blocked by WAY100635, as well as by the CB1 receptor antagonist AM251, supporting the hypothesis that CBD’s effects could also involve indirect activation of CB1 receptors by increasing AEA levels, which could in turn modulate the activity of 5HT1A receptors. In agreement with this assumption, the antidepressant-like effects induced by AEA were blocked by local administration of a 5HT1A antagonist [80]. Moreover, genetic deletion of the *FAAH* gene, which codes for the protein that breaks down AEA, also produced antidepressant-like effects paralleled by altered 5-HT transmission and postsynaptic 5-HT1A activation [83].

### 4.3. Putative Epigenetic Mechanisms Underlying CBD’s Antidepressant-Like Properties

As for CBD’s anxiolytic effects, the molecular mechanisms underlying CBD’s antidepressant-like properties may involve epigenetic mechanisms that include DNA methylation, histone modifications, and the regulation of miRNA expression.

#### 4.3.1. CBD and DNA Methylation

Reports indicating that CBD may target the regulation of DNA methyltransferases, such as Dnmt1 and Dnmt3a [52,54], are of particular relevance for mood disorders. Specifically, DNA methylation is considered to play an important role in the development of depression [84]. Moreover, the expression of DNMTs has been linked to the antidepressant-like effects of escitalopram, imipramine, and amitriptyline [85,86,87]. Similarly, bipolar patients have also been found to present with alterations in DNA methyltransferases, particularly DNMT1, and mood stabilizers have been found to affect DNA methylation levels [88]. Findings suggesting the presence of similar downstream epigenetic effects of CBD and antidepressants provide further support to the hypothesis that CBD’s antidepressant-like properties are mediated by increased serotonergic activity through 5-HT1A receptors [72,73,76,80]. In line with this assumption, a recent mouse study found that pretreatment with CBD (10 mg/kg, i.p.), or DNA methylation inhibitors produced antidepressant-like effects in the FST [89]. The latter finding parallels the antidepressant-like effect of 5-HT1A agonists found in the same test [90]. Interestingly, however, the CBD study reported opposing effects of stress on DNA methylation in the hippocampus versus the prefrontal cortex [89]. Specifically, stress induced by FST increased the levels of both global DNA methylation and DNMT activity in the hippocampus, whereas the opposite pattern—i.e., decreased DNA methylation and DNMT activity—was observed in the prefrontal cortex. Importantly, pretreatment with CBD prevented the latter stress-induced epigenetic changes from happening in both the hippocampus and the PFC [89]. These data raise the possibility that the antidepressant-like properties of CBD could involve serotonergic-induced modulation of DNMTs which, in turn, lead to DNA methylation changes that counteract affective pathophysiologies.

#### 4.3.2. CBD and Histone Modifications

The role of histone acetylation and methylation has been extensively studied in preclinical models of depression [91]. Importantly, there is evidence linking depression-like behaviors to histone modifications found to be affected by CBD (20 mg/kg, i.p.), including H3K9ac, H3K14ac, H3K4me3, H3K9me2, and H3K27me3 [59,60]. For example, decreased hippocampal levels of H3K9ac have been associated with depression-like behaviors that are induced by chronic unpredicted stress, prenatal stress, or drug exposure and—importantly—these changes have been reversed by antidepressant treatment with venlafaxine [92,93,94]. The levels of H3K14ac have also been linked to depression-like behaviors in a brain region-dependent manner. Specifically, H3K14ac was found to be persistently increased in the mouse nucleus accumbens following chronic social defeat stress and in the brain of depressed patients following postmortem examination [95]. However, opposite to the effects of chronic social defeat in the nucleus accumbens, a persistent decrease in H3K14ac levels was found in the hippocampus, which was reversed by a chronic antidepressant treatment with fluoxetine [96]. Significant alterations in H3K9me2 levels, reversed by chronic antidepressant treatment, have also been found in the nucleus accumbens of mice exposed to chronic social defeat or chronic social isolation [97]. Moreover, mice with a history of early-life stress showed enhanced susceptibility to social defeat stress in adulthood and had genome-wide alterations in H3K4me3 levels in the prefrontal cortex [98]. Changes in H3K4me3 levels at synapsin genes have also been found in postmortem brain samples from individuals with major depression and bipolar disorder [99]. Finally, rodent studies have also linked H3K9me2 and H3K27me3 levels to depression-like behaviors that are induced by early-life stress, social defeat stress, or maternal separation [100,101,102]. These findings provide initial clues to the underlying histone modifications that may mediate the antidepressant action of CBD and warrant further examination in rodent models of depression.

#### 4.3.3. CBD and miRNAs

Alterations in the levels of miRNAs have been described in both brain and peripheral (blood) tissues of patients with depression and bipolar disorder [103,104,105]. The effects of CBD on the expression of certain miRNAs may also provide a working hypothesis to its underlying antidepressant-like molecular mechanisms. For instance, in the BV-2 microglial study, CBD treatment (10 μM) affected the expression of miR-146a and miR-155 [65]. Studies on major depression have found the downregulation of miR-146a in the blood of patients who respond to antidepressant treatment [106]. However, the reverse pattern of miR-146a expression was recently observed in a study using monocytes of depressed patients on antidepressants [107]. With regard to miR-155, knockout mice exhibited reduced depression-like behaviors [67]. However, peripheral miR-155 levels were found to be lower in depressed patients and increased following antidepressant treatment [107]. In the BV-2 microglial study, CBD (10 μM) upregulated miR-34a [65], which has also been implicated in both unipolar and bipolar depression. Specifically, miR-34a has been found to be increased in the cerebrospinal fluid and serum of individuals with major depressive disorder [108]. Moreover, miR-34a has been found to target the bipolar risk genes ankyrin-3 and voltage-dependent L-type calcium channel subunit beta-3 and was linked to impaired neuronal differentiation and morphology [109]. Collectively, although some of CBD’s effects on miRNA expression appear to overlap with those on miRNAs that are dysregulated in mood disorders, it should be noted that the findings so far originate from microglial cell cultures only. Thus, in vivo studies are warranted in order to provide a better understanding of CBD’s effects on miRNA expression, particularly in brain regions involved in affective psychopathology.

## 5. Conclusions

CBD’s therapeutic potential has been investigated in a number of neuropsychiatric diseases and pathological conditions. In the present preclinical review, we provided an overview of behavioral and neuropharmacological studies that evaluated CBD as an anxiolytic- and antidepressant-like compound. Moreover, we outlined evidence suggesting that CBD’s therapeutic-like properties may involve epigenetic mechanisms that include DNA methylation, histone modifications, and the regulation of miRNA expression. Collectively, and given CBD’s safety profile, these studies support the continued evaluation of CBD as a promising new agent in the treatment of anxiety and mood disorders. However, future studies are still warranted to elucidate CBD’s precise pharmacodynamic profile and epigenetic mechanisms of action.

## Figures and Tables

**Table 1:** CBD effects in animal models of anxiety.

Animal Model	Animal	Dose/Route of Administration	Effect	Mechanism	Reference
EPM	Rats	2.5, 5, 10 mg/kg, acute, i.p.	Anxiolytic	Not investigated	[16]
EPM	Mice	2.5, 5, 10, 50 mg/kg, acute, i.p.	Anxiolytic	Not investigated	[17]
SI	Mice	1 mg/kg, acute, i.p.	Anxiolytic	Not investigated	[18]
VCT	Rats	10 mg/kg, acute, i.p.	Anxiolytic	Not investigated	[19]
Restrain stress	Rats	10 mg/kg, acute, i.p.	Anxiolytic	5-HT1A receptors	[20]
Light- dark test	Rats	5 mg/kg, acute, i.p.	Anxiolytic	Not investigated	[21]
EPM	Rats	30 nmol/µL; intra-dPAG	Anxiolytic	5-HT1A receptors	[22]
ETM	Rats	30 and 60 nmol/µL; intra-dPAG	Anxiolytic	5-HT1A receptors	[23]
EPM/VCT	Rats	1 µg/µL; intra-CeA	Anxiolytic	Not investigated	[24]
EPM/VCT	Rats	30 nmol/µL; intra-BNST	Anxiolytic	5-HT1A receptors	[25]
CFC	Rats	30 and 60 nmol/µL; intra-BNST	Anxiolytic	5-HT1A receptors	[26]
Restrain stress	Rats	30 nmol/µL; intracisternal	Anxiolytic	Not investigated	[27]
CFC	Rats	30 nmol/µL; intra-PL	Anxiolytic	5-HT1A receptors	[28]
EPM	Rats	30 nmol/µL; intra-PL	Anxiogenic	5-HT1A receptors	[28]
EPM	Rats	60 nmol/µL; intra-dPAG + Capsazepine	Anxiolytic	TRPV1 receptors	[29]
ETM	Rats	5 mg/kg/daily/21 days; i.p.	Anxiolytic	5-HT1A receptors	[30]
Chronic Stress	Mice	30 mg/kg; /daily/14days; i.p.	Anxiolytic	Hippocampal Neurogenesis; CB1 receptors	[31]
CER	Rats	10 mg/kg/daily/14 days; i.p.	Anxiogenic	BDNF ↓ Trk B ↓	[32]

Abbreviation: EPM: elevated plus maze; SI: social interaction; VCT: Vogel’s conflict test; ETM: elevated T-maze; CFC: contextual fear conditioning; CER: conditioned emotional response; dPAG: dorsal portions of the periaqueductal gray matter; CeA: central nucleus of amygdala; BNST: bed nucleus of the stria terminalis; PL: prelimbic prefrontal cortex; i.p.: intraperitoneal; BDNF: brain-derived neurotrophic factor; TrkB: tyrosine kinase B receptor; ↑: increase; ↓: decrease.

**Table 2:** CBD effects in animal models of depression.

Animal Model	Animal	Dose/Route of Administration	Effect	Mechanism	Reference
FST	Mice	30 mg/kg, acute, i.p.	Antidepressant	5-HT1A receptors; BDNF *unaltered*	[72]
FST	Mice	7–30 mg/kg, acute, i.p.	Antidepressant	↑ BDNF	[79]
FST	Mice	30 mg/kg, acute, i.p. ≈ 7 mg/kg + 5 mg/kg fluoxetine, acute, i.p.	Antidepressant	5-HT levels	[73]
FST	Rats	30 mg/kg i.p. per day for 14 days	Antidepressant	↑ BDNF	[75]
OBX	Rats	50 mg/kg, acute, i.p.	Antidepressant	↑ 5-HT; ↑ Glu	[76]
OBX	Rats	50 mg/kg per day for 3 days + 10 mg/kg per day for 11 days	Antidepressant	5-HT1A receptors	[76]
WKY	Rats	30 mg/kg, acute, orally	Antidepressant	Not investigated	[77]
FSL	Rats	30 mg/kg, acute, orally	Antidepressant	Not investigated	[78]
FST	Rats	45 and 60 nmol/µL intra-mPFC	Antidepressant	5-HT1A receptors; CB1 receptors	[80]

Abbreviation: FST: forced swimming test; OBX: olfactory bulbectomy mouse model of depression; WKY: Wistar Kyoto rats; FSL: Flinders Sensitive Line rats; mPFC: medial prefrontal cortex; 5-HT: serotonin; Glu: glutamate; i.p.: intraperitoneal; BDNF: brain-derived neurotrophic factor ↑: increase.
